# Differentiating the Cognitive Profile of Schizophrenia from That of Alzheimer Disease and Depression in Late Life

**DOI:** 10.1371/journal.pone.0010151

**Published:** 2010-04-12

**Authors:** Christina Ting, Tarek K. Rajji, Zahinoor Ismail, David F. Tang-Wai, Nina Apanasiewicz, Dielle Miranda, David Mamo, Benoit H. Mulsant

**Affiliations:** 1 Geriatric Mental Health Program, Centre for Addiction and Mental Health, Toronto, Ontario, Canada; 2 Department of Psychiatry, University of Toronto, Toronto, Ontario, Canada; 3 Department of Psychiatry, University of Calgary, Calgary, Alberta, Canada; 4 Division of Neurology, University of Toronto, Toronto, Ontario, Canada; Chiba University Center for Forensic Mental Health, Japan

## Abstract

**Background:**

To compare the cognitive profile of older patients with schizophrenia to those with other neuropsychiatric disorders assessed in a hospital-based memory clinic.

**Methods:**

Demographic, clinical, and cognitive data of all patients referred to the memory clinic at the Centre for Addiction and Mental Health between April 1, 2006 and August 15, 2008 were reviewed. We then identified four groups of older patients with: (1) late-life schizophrenia (LLS) and no dementia or depression (DEP); (2) Alzheimer's disease (AD); (3) DEP and no dementia or LLS; (4) normal cognition (NC) and no DEP or LLS.

**Results:**

The four groups did not differ in demographic data except that patients with AD were about 12 years older than those with LLS. However, they differed on cognitive tests even after controlling for age. Patients with LLS were impaired on most cognitive tests in comparison with patients with NC but not on recalling newly learned verbal information at a short delay. They experienced equivalent performance on learning new verbal information in comparison with patients with AD, but better performance on all other tests of memory, including the ability to recall newly learned verbal information. Finally, they were more impaired than patients with DEP in overall memory.

**Conclusions:**

Patients with LLS have a different cognitive profile than patients with AD or DEP. Particularly, memory impairment in LLS seems to be more pronounced in learning than recall. These findings suggest that cognitive and psychosocial interventions designed to compensate for learning deficits may be beneficial in LLS.

## Introduction

Cognitive deficits are common in patients with schizophrenia or depression (DEP) including in late life [1], [2]. In patients with schizophrenia, specific deficits, such as memory impairment, are reliable predictors of function [3], and are considered critical targets for the development of novel pharmacological or psychosocial therapies [4]. Thus, the identification of the cognitive deficits that are specific to late-life schizophrenia (LLS) in contrast to late-life DEP or other neuropsychiatric disorders (e.g., Alzheimer's disease (AD)) is critical to facilitate diagnosis and support the development of specific treatment interventions (“personalized treatment”). For example, an effective cognitive intervention can be tailored to a specific cognitive function that is more impaired in LLS than in DEP. A similar selective approach could be applied to combinations of disorders (e.g., LLS and DEP or LLS and AD). To our knowledge, only a few published studies compared patients with LLS to those with AD [2], [5], [6], [7], [8], and none to those with DEP. Furthermore, and except for Heaton et al [2], these studies reported on a global cognitive test (e.g., Mini-Mental State Examination or Dementia Rating Scale total score) or a brief cognitive battery (e.g., 3 tests from the Consortium to Establish a Registry for Alzheimer's Disease) which limited their ability to distinguish specific cognitive deficits in different cognitive domains. Thus, we took advantage of the comprehensive cognitive assessment that is administered to patients referred to hospital-based memory clinic and we compared the cognitive profiles of patients with LLS to those of patients with AD, DEP, or “normal cognition” (NC).

## Methods

### Setting and Subjects

The Centre for Addiction and Mental Health (CAMH) in Toronto, Canada is a unique and large academic specialty hospital that provides psychiatric care (including care to patients with dementia) to an urban catchment area and serves as a referral center for a large suburban and rural population. This analysis was based on a review of the health records of all patients referred for a consultation to the memory clinic at CAMH between April 1, 2006 and August 15, 2008. These patients had undergone a comprehensive assessment including a neurological evaluation (by D.F.T-W.), a psychiatric examination (by Z.I.), and cognitive testing (by N.A.). Diagnoses were ascertained using a consensus process that was mainly guided by the neurological and psychiatric assessments. However, patients with schizophrenia had this diagnosis established by the referring source and then confirmed by the psychiatrist (Z.I.) at the memory clinic. Since this study was based on the review of health records of patients who were already assessed at the memory clinic, no informed consent from the patients was obtained and the data were analyzed anonymously. The Centre for Addiction and Mental Health Research Ethics Board waived the need for consent and approved the study.

### Data Abstraction

Charts were reviewed and data abstracted by two investigators (C.T., and T.K.R.) using a standardized form. The following tests were recorded and analyzed: age, sex, education, neuropsychiatric diagnoses, residential type, and cognitive data. The cognitive battery used at the memory clinic includes the following tests: Animal Fluency [9], Boston Naming Test [10], Clock drawing test – Freedman scale (Clock) [11], California Verbal Learning Test II- Short Form (CVLT) [12], Dementia Rating Scale-2 (DRS) [13], FAS Letter Fluency (FAS) [9], Luria Alternating Diagrams [14], Mini-Mental Status Examination (MMSE) [15], Trail Making Test A and B (TMA, TMB) [16], and Wisconsin Card Sorting Test – 64 (WCST) [17]. Following McBride et al [7], we calculated two CVLT Saving Scores: one for Short Delay Free Recall and another for Long Delay Free Recall. We generated these scores by dividing the number of correct responses at Short Delay Free Recall (or Long Delay Free Recall) by the number of correct responses at the last trial of acquisition of CVLT, and multiplying the answer by 100.

### Data Analysis

After completing the clinical and cognitive assessments, a consultation report was generated for each referred patient. The report included a neuropsychiatric diagnostic formulation that was based on the application of the Diagnostic and Statistical Manual of Mental Disorders, Fourth Edition, Text Revision criteria to the data obtained during these assessments. We used this diagnostic formulation to identify (1) a group of patients with LLS and without a diagnosis of dementia; from the rest of the patients, we created three comparison groups: (2) a group of patients with AD and without another mental disorder; (3) a group of patients with DEP and no dementia; and (4) a group of patients without a neuropsychiatric disorder and with NC. The latter group (NC) consisted of individuals who were referred to the clinic for memory concerns or complaints and who were ascertained to be cognitively intact based on their clinical and cognitive assessments. First, we characterized descripitively the four groups of patients. Then, we compared the demographic, clinical, and cognitive characteristics of the four groups using one-way analyses of variance (ANOVA). When differences were found, we used post-hoc comparisons with Bonferroni's corrections to correct for multiple comparisons. Cohen's effect sizes (d's) were calculated for differences between LLS and the other three groups on all cognitive tests. Data analyses were conducted using SPSS 15.0 for Windows.

## Results

### Diagnostic groups

One hundred twenty four individual patients were assessed at the memory clinic during the study period. Five patients were excluded due to language barriers preventing the completion of significant portions of the comprehensive assessment. Of the remaining 119 patients, 52 patients were excluded for the following diagnoses: cognitive disorder not-otherwise specified: N = 10; alcohol-related disorder: N = 5; schizophrenia and dementia N = 5; bipolar disorder: N = 4; depression and dementia: N = 4; frontal lobe degeneration/syndrome or semantic dementia: N = 4; mental retardation: N = 4; dementia with Lewy bodies: N = 3; corticobasal degeneration syndrome: N = 2; dementia not-otherwise specified: N = 2; dementia with multiple etiologies: N = 2; mild cognitive impairment with parkinsonism: N = 2;; Alzheimer's disease with primary progressive aphasia: N = 1; asymmetrical cortical degeneration: N = 1; depression and dementia with Lewy bodies: N = 1; depression and possible neurodegenerative disorder: N = 1;. vascular dementia: N = 1. The remaining 67 patients were classified among the groups of interest as follows: (1) LLS without dementia: N = 25; (2) AD: N = 15; (3) DEP: N = 15; (4) NC: N = 12.

### Demographic and clinical characteristics

The subjects' characteristics data are summarized in Table S1. Patients with LLS were not different from the other groups with respect to sex distribution, education, race, community living status, or number of active medical problems. However, on average, they were 12 years younger than those with AD.

### Cognitive characteristics

**Figure 1:**
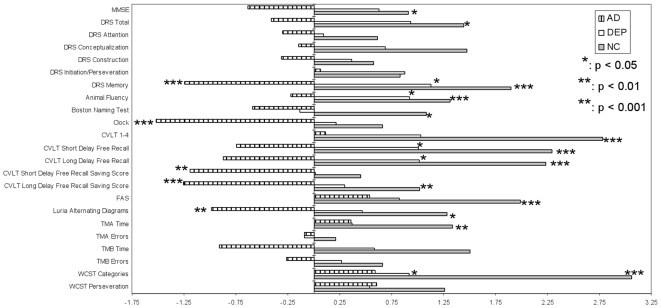
Comparison of Cognitive Performances of Patients with Alzheimer's Disease, Depressive Disorder, or Normal Cognition to those with Late-Life Schizophrenia. Cohen's effect sizes have been calculated and plotted for the different cognitive tests comparing performances of patients with AD, DEP, or NC to those with LLS (who constitute the reference group). Positive effect sizes indicate that patients with AD, DEP, or NC perform numerically better than patients with LLS. Negative effect sizes indicate that the opposite. * indicates that the difference is statistically significant (p<0.05) after correcting for multiple comparisons. ** indicates that the difference is statistically significant (p<0.01) after correcting for multiple comparisons. *** indicates that the difference is statistically significant (p<0.001) after correcting for multiple comparisons. AD: Alzheimer's disease; Clock: Clock drawing test – Freedman scale; CVLT: California Verbal Learning Test II- Short Form; DEP: depressive disorder; DRS: Dementia Rating Scale-2; FAS: FAS Letter Fluency; MMSE: LLS: late-life schizophrenia; Mini-Mental Status Examination; NC: normal cognition; TMA and TMB: Trail Making Test A and B, respectively; WCST: Wisconsin Card Sorting Test – 64.

The cognitive characteristics of the four groups are presented in Table S1 and Figure 1. After Bonferroni's correction, the patients with LLS performed significantly better than those with AD on DRS Memory, CLOCK, CVLT Short Delay Free Recall Saving Score and Long Delay Free Recall Saving Score, and Luria Alternating Diagrams; performed significantly worse than those with DEP on DRS Memory, Animal Fluency, CVLT Short Delay Free Recall, and Long Delay Free Recall, and WCST Categories; and worse than those with NC on MMSE, DRS Total and Memory, Animal Fluency, Boston Naming Test, CVLT 1-4, Short Delay Free Recall, Long Delay Free Recall and Long Delay Free Recall Saving Score, FAS, Luria Alternating Diagrams, TMA Time, and WCST Categories.

We also performed the same analyses excluding subjects above the age of 50 (2 subjects with LLS and 1 subject with NC). The only significant change is that the difference between patients with LLS and those with DEP on FAS became significant (p = 0.042).

## Discussion

Using a comprehensive cognitive assessment, we compared the cognitive profiles of patients seen at a hospital-based memory clinic with LLS, AD, DEP, or NC. Three main findings emerge from this analysis: (1) patients with LLS were impaired on most cognitive tests in comparison with patients with normal cognition (NC); (2) patients with LLS experienced impairment on learning new verbal information that was equivalent to that experienced by patients with AD, but a significantly smaller impairment in their ability to retrieve this newly learned verbal information; (3) patients with LLS were more impaired than patients with DEP on all tests of memory except the ability to retrieve newly learned verbal information.

These findings need to be considered in the context of the strengths and the limitations of this study. First, because this memory clinic is based in a large psychiatric hospital, more than half of the patients had primary psychiatric diagnoses (e.g., alcohol-related disorder, LLS, Bipolar disorder, DEP) usually not represented in a community-based memory clinic. Due to this heterogeneity in diagnosis, some patients had to be excluded from the analysis and the four groups that were included were small. This reduced the power to detect statistically significant differences on specific tests, especially those of executive function (e.g., DRS Initiation/Perseveration subscales, and WCST). Still, large and highly significant differences on most tests were detected between patients with LLS and those without a psychiatric diagnosis who were referred by their primary care physicians due to memory or cognitive complaints and found to have normal cognition. Second, the deficits identified in these patients with LLS who were referred to the clinic *because* of “memory complaints” may not generalize to the larger population of older persons with LLS. This applies in particular to the differences in the domain of memory. Nevertheless, such a bias towards memory impairment should not have affected the differences observed between the four studied groups since *all* the patients seen in the clinic were referred because of “memory complaints.” Third, patients with AD were about 12 years older than patients with LLS. Thus, age may have contributed to the more severe memory impairment seen in patients with AD. However, our findings in memory did not change significantly when we limited our analysis to patients age 50 or above. Further, a lesser memory impairment in LLS compared to AD has also been reported in age-matched patients [5], [7]. Also, such a confound would not explain the equivalent impairments in learning new verbal information but smaller impairment in LLS compared to AD in the ability to retrieve newly learned information. Finally, more than 80% of our patients with LLS were community-dwellers and the current literature on such patients suggests cognitive stability rather than decline with age [18].

Notwithstanding the above limitations, our findings confirm and extend our current understanding of cognition in LLS. The ranking of the groups in terms of overall memory impairment (AD < LLS < DEP) and the large effect sizes observed when patients with LLS were compared with those with DEP and AD has significant therapeutic and functional implications: interventions to enhance cognition in LLS should have a special focus on memory. Furthermore, memory correlates significantly with everyday function such as hygiene, safety, cooking, and community utilization in the geriatric population [19]. This suggests that interventions enhancing memory could have a significant functional impact, but that the impact may be different in patients with different neuropsychiatric disorders.

In contrast to comparable deficits in learning new verbal information (CVLT 1-4), patients with LLS experienced significantly smaller deficits than those with AD in retrieving the newly learned information (CVLT Short and Long Delay Free Recall Saving Scores). In fact, they experienced comparable performance to those with NC in recalling newly learned verbal information at a short delay. These findings in LLS are consistent with previous reports among mid to late life patients with schizophrenia, suggesting that the memory impairment associated with LLS is secondary to poor registration or organization of new information and not impaired retrieval of learned information as in AD [2], [20], [21]. This is relevant to the understanding of the cognitive pathology in LLS compared to AD, and to the design of cognitive interventions. Facilitating the encoding of new information is likely to have a more meaningful and clinically significant impact in LLS than in AD (because the chance of retrieving encoded information is higher in LLS than AD).

Other than memory, patients with LLS performed better than patients with AD on the CLOCK, a test of visuospatial ability (amongst other abilities). Thus, like recall, visuospatial ability appears to be another cognitive function that can be used in differentiating cognitively impaired patients with LLS from those with AD. Furthermore, visuospatial ability has also been associated with function and has been reported to decline in a longitudinal study of subjects with LLS who were older than our patients [22], [23]. Thus, it can also be a target for interventions to enhance their cognition.

Finally, as expected patients with LLS were impaired in executive function compared to patients with DEP or NC on one test (WCST Categories). However, no such impairment was observed in other tests (e.g., TMB). Small sample sizes are likely contributing to such inconsistency.

### Conclusion

In conclusion, patients with LLS and no dementia can be distinguished from patients with other neuropsychiatric disorders by their distinct cognitive profile, in particular their relatively intact recall of learned information and visuospatial ability. This finding may be useful in the development of interventions to enhance the cognition of patients with LLS.

## Supporting Information

Table S1 LLS: late-life schizophrenia; AD: Alzheimer's disease; DEP: depressive disorder; NC: normal cognition. -- All results presented as mean scores (SD) unless specified otherwise. *: group(s) significantly different from LLS after Bonferroni's correction **: duration of illness available for 13 patients with LLS, 1 patient with DEP, and no one with AD or NC [n]  =  number of individuals contributing to the mean {n}  =  Cohen's effect size of the comparison between AD, DEP, or NC group with LLS group.
(0.09 MB DOC)
