# Supplementary material for: Differentiating the Cognitive Profile of Schizophrenia from That of Alzheimer Disease and Depression in Late Life
Source: PLoS One. 2010 Apr 12;5(4):e10151. doi: 10.1371/journal.pone.0010151 (PMC2853579; doi:10.1371/journal.pone.0010151)
Supplement: Table S1 — LLS: late-life schizophrenia; AD: Alzheimer's disease; DEP: depressive disorder; NC: normal cognition. -- All results presented as mean scores (SD) unless specified otherwise. *: group(s) significantly different from LLS after Bonferroni's correction **: duration of illness available for 13 patients with LLS, 1 patient with DEP, and no one with AD or NC [n] = number of individuals contributing to the mean {n} = Cohen's effect size of the comparison between AD, DEP, or NC group with LLS group. (0.09 MB DOC) [file pone.0010151.s001.doc]

**Table S1: Subjects’ Demographic and Cognitive Characteristics**

|  | LLS | AD | DEP | NC | Statistics; p-value; Bonferroni’s  correction(s)* |
| --- | --- | --- | --- | --- | --- |
| N | 25 | 15 | 15 | 12 | -- |
| Female – N (%) | 11 (44.0%) | 7 (46.7%) | 8 (53.3%) | 7 (58.3%) | χ ^2^ = 3.28; p=0.350 |
| Caucasian – N/N of subjects for whom data is available (%) | 18/23 (78.3%) | 14/15 (93.3%) | 10/15 (66.6%) | 10/12 (83.3%) | χ ^2^ = 3.41; p=0.333 |
| Community-dwellers –  N/N of subjects for whom data is available (%) | 18/22 (81.8%) | 14/14 (100.0%) | 12/12 (100.0%) | 7/7 (100.0%) | χ ^2^ = 6.35; p=0.096 |
| Age (years) | 61.2 (9.3) | 73.5 (11.3) | 65.7 (9.2) | 62.0 (10.2) | F(3,63)=5.21; p=0.003;  AD |
| Education (years) | 11.2 (3.4) | 10.9 (4.4) | 12.3 (4.1) | 14.2 (3.3) | F(3,62)=2.17; p=0.100 |
| Duration of Illness (years)** | 30.9 (10.5) | -- | 27.0 | -- | -- |
| Number of active medical problems | 3.2 (1.9) | 3.5 (2.6) | 3.8 (2.0) | 3.9 (2.5) | F(3,58)=0.35; p=0.791 |
| MMSE | 24.7 (5.5) [25] | 21.3 (5.1) [15] {-0.63} | 27.6 (2.4) [15] {0.63} | 28.9 (1.4) [12] {0.91} | F(3,63)=8.52; p=0.000; NC |
| DRS Total score (DRS Total) | 114.3 (15.9) [16] | 107.1 (19.4) [13] {-0.41} | 127.0 (8.6) [10] {0.93} | 135.0 (5.8) [5] {1.44} | F(3,40)=5.86; p=0.002; NC |
| DRS Attention subscale (DRS Attention) | 34.7 (2.4)  [16] | 33.8 (3.6) [13] {-0.30} | 34.9 (1.7) [10] {0.09} | 36.0 (0.9) [6] {0.61} | F(3,41)=1.09; p=0.366 |
| DRS Conceptualization subscale (DRS Conceptualization) | 29.9 (6.1) [16] | 28.8 (9.6) [13] {-0.14} | 33.7 (4.1) [10] {0.69} | 37.8 (1.0) [6] {1.48} | F(3,41)= 3.14; p=0.035 |
| DRS Construction subscale (DRS Construction) | 5.5 (1.0) [16] | 5.2 (0.9)  [13] {-0.31} | 5.8 (0.4) [10] {0.36} | 6.0 (0.0)  [6] {0.58} | F(3,41)= 1.57; p=0.212 |
| DRS Initiation/Perseveration subscale (DRS Initiation/Perseveration) | 26.9 (6.3)  [16] | 27.3 (5.9) [13] {0.07} | 31.6 (3.3) [10] {0.87} | 32.0 (5.4) [5] {0.83} | F(3,40)=2.29; p=0.093 |
| DRS Memory subscale (DRS Memory) | 17.0 (4.1)  [16] | 12.1 (3.6) [13] {-1.24} | 21.0 (2.2) [10] {1.13} | 23.8 (0.8) [6] {1.90} | F(3,41)=21.81; p=0.000; AD, DEP, NC |
| Animal fluency | 10.2 (4.7) [22] | 9.2 (3.9)  [12] {-0.22} | 14.5 (4.7) [13] {0.92} | 16.4 (5.0) [11] {1.31} | F(3,54)=7.31; p=0.000; DEP, NC |
| Boston Naming Test (%) | 72.3 (23.5) [22] | 58.3 (24.7) [12]{-0.59} | 69.1 (23.8) [15]{-0.14} | 93.6 (5.9) [11] {1.08} | F(3,56)=5.25, p=0.003; NC |
| Clock Drawing Test (Clock) | 13.1 (2.2) [20] | 8.2 (4.4)  [13] {-1.52} | 13.6 (2.0) [13] {0.21} | 14.4 (1.0) [10] {0.66} | F(3,52)=13.50; p=0.000; AD |
| CVLT Trials 1-4 (CVLT 1-4) (T-score) | 26.8 (12.4)  [23] | 28.1 (8.4) [12] {0.11} | 39.5 (12.0) [12] {1.03} | 60.2 (10.9) [9] {2.78} | F(3,58=24.55) p=0.000; NC |
| CVLT Short Delay Free Recall (T-score) | 33.2 (8.9)  [25] | 27.3 (5.6) [13] {-0.74} | 42.7 (10.5) [13] {1.01} | 55.9 (12.0) [11] {2.29} | F(3,58)=22.51 p=0.000; DEP; NC |
| CVLT Long Delay Free Recall (T-score) | 36.0 (8.4) [24] | 29.2 (6.4) [13] {-0.87} | 45.0 (9.8) [13] {1.01} | 58.2 (12.9) [11] {2.23} | F(3,53)=6.47, p=0.001;  DEP; NC |
| CVLT Short Delay Free Recall Saving Score (%) | 84.8 (30.9) [22] | 50.5 (24.6) [13] {-1.19} | 85.2 (27.4) [12] {0.01} | 98.2 (26.8) [10] {0.45} | F(3, 53)=6.47, p=0.001;  AD |
| CVLT Long Delay Free Recall Saving Score (%) | 67.7 (40.2)  [22] | 24.6 (20.1) [13] {-1.25} | 78.3 (26.7) [12] {0.29} | 106.0 (30.9) [10] {1.02} | F(3, 53)=12.86; p=0.000;  AD; NC |
| FAS Letter Fluency (FAS) | 18.7 (9.6) [23] | 24.2 (11.1) [11] {0.54} | 27.1 (11.0) [12] {0.83} | 38.0 (9.8) [11] {1.99} | F(3,53)=8.95, p=0.000; NC |
| Luria Alternating Diagrams | 4.5 (1.4) [22] | 2.7 (2.3)  [14] {-0.98} | 5.1 (1.0) [15] {0.48} | 6.0 (0.0) [12] {1.29} | F(3,59)=12.00, p=0.000; AD, NC |
| Trails Making A – Time to completion (TMA Time) | 93.5 (47.8) [22] | 77.9 (31.6) [11] (0.36} | 73.1 (65.1) [15] {0.37} | 39.2 (20.8) [12] {1.34} | F(3,56)=3.53; p=0.021; NC |
| Trails Making A – Number of errors (TMA Errors) | 0.5 (1.1)  [22] | 0.6 (1.1)  [13] {-0.09} | 0.6 (1.1) [15]{-0.09} | 0.3 (0.6) [12] {0.21} | F(3,58)=0.20, p=0.898 |
| Trails Making B – Time to completion (TMB Time) | 225.0 (95.6) [18] | 389.6 (363.2)  [5] {-0.91} | 165.0 (115.2) [11] {0.58} | 93.3 (73.2) [12] {1.50} | F(3,42)=5.43, p=0.003 |
| Trails Making B – Number of errors (TMB Errors) | 1.6 (2.5) [16] | 2.2 (1.1)  [5] {-0.26} | 1.0.(1.7)  [9] {0.27} | 0.3 (0.5) [11] {0.66} | F(3,37)=1.81, p=0.162 |
| Wisconsin Card Sorting Test Categories Completed (WCST Categories) | 1.1 (1.2)  [15] | 1.7 (0.8)  [6] {0.59} | 2.5 (1.8) [13] {0.92} | 4.1 (0.6)  [9] {3.06} | F(3,39)=10.72, p=0.000; DEP, NC |
| Wisconsin Card Sorting Test Perseverative Response (WCST Perseveration) | 25.6 (17.1) [8] | 16.7 (5.7)  [4] {0.61} | 17.2 (14.1)  [8] {0.54} | 7.5 (1.7)  [4] {1.26} | F(3,20)=1.71, p=0.196 |

LLS: late-life schizophrenia; AD: Alzheimer’s disease; DEP: depressive disorder; NC: normal cognition. -- All results presented as mean scores (SD) unless specified otherwise.

*: group(s) significantly different from LLS after Bonferroni’s correction

**: duration of illness available for 13 patients with LLS, 1 patient with DEP, and no one with AD or NC

[n] = number of individuals contributing to the mean

{n} = Cohen’s effect size of the comparison between AD, DEP, or NC group with LLS group
